# The impact of Withania somnifera supplementation on sleep quality and stress level in women with polycystic ovary syndrome: preliminary results

**DOI:** 10.1192/j.eurpsy.2026.11924

**Published:** 2026-07-31

**Authors:** K. Stańczyk, M. Stuss, O. Gawlik-Kotelnicka, K. Szmigielska, K. Wielemborek-Musiał, A. Lipert

**Affiliations:** 1 Department of Sports Medicine; 2 Department of Endocrine Disorders and Bone Metabolism; 3 Department of Affective and Psychotic Disorders; 4 Department of Coordinated Care; 5 Department of Preventive Medicine, Medical University of Lodz, Lodz, Poland

## Abstract

**Introduction:**

Polycystic ovary syndrome (PCOS) is the most prevalent endocrine disorder in reproductive-age women, with a prevalence estimated at 4–20%. PCOS patients are at a higher risk of developing sleep disorders and experience greater stress levels than the general population. *Withania somnifera* has been identified as a candidate for supporting the treatment of these disorders, due to evidence indicating its capacity to enhance sleep quality by modulating γ-aminobutyric acid signaling, and to reduce stress by withanolides activity.

**Objectives:**

To assess the impact of 3-month *Withania somnifera* (Ashwagandha) supplementation on sleep quality and stress level in PCOS women.

**Methods:**

The study involved data from 18 patients of the Endocrinology Outpatient Clinic of the Central Clinical Hospital of the Medical University of Lodz, aged 21–34 years. The intervention comprised the administration of dietary supplements (study group: 2000 IU of cholecalciferol, 400 μg of folic acid, 400 μg of 5-methyltetrahydrofolate, and standardized *Withania somnifera* extract for 10 mg of withanolides per day; control group: 2000 IU of cholecalciferol, 400 μg of folic acid, and 400 μg of 5-methyltetrahydrofolate per day) over a period of 3 months. The Pittsburgh Sleep Quality Index (PSQI) and the Perceived Stress Scale (PSS-10), as well as socio-demographic and health-related data questionnaire were used to conduct a survey.

**Results:**

PCOS was diagnosed on average 3.50±3.64 years prior to participation in the study. More than half of the women had a normal body mass index. In the initial assessment (N=18), the mean PSQI and PSS-10 scores were 6.72±3.75 (p=0.005) and 20.50±5.51 (p=0.390), respectively. The analysis revealed that nearly half of the patients demonstrated poor sleep quality, and over ¾ had moderate (f=0.28) or high stress levels (f=0.56). The baseline data indicated a significant, positive and moderate correlation between sleep quality and stress levels (r=0.53, p=0.024). The mean PSQI and PSS-10 scores in the pre-intervention and post-intervention measurements were 8.67±2.52 vs. 8.33±2.52 and 21.67±5.51 vs. 21.33±11.06 in the study group (n=3), and 5.00±1.00 vs. 4.33±0.58 and 21.00±7.21 vs. 17.00±6.24 in the control group (n=3). Following the intervention, some effect on sleep quality (Me: 9.00, IQR: 5.00 and Me: 8.00, IQR: 5.00 vs. Me: 5.00, IQR: 2.00 and Me: 4.00, IQR: 1.00) and stress levels (Me: 22.00, IQR: 11.00 and Me: 20.00, IQR: 22.00 vs. Me: 19.00, IQR: 14.00 and Me: 15.00, IQR: 12.00) was observed.

**Conclusions:**

The correlation between sleep quality and stress level was observed, which justifies the necessity of providing psychiatric and psychological support to PCOS patients. A trend toward improved sleep quality parameters and reduced stress levels was observed in the study group. Further research is required to evaluate the results of the intervention.

**Disclosure of Interest:**

None Declared

